# Sex Steroid Modulators and the Development of Buschke-Lowenstein Tumor: A Case Report of an Immunocompetent Patient

**DOI:** 10.7759/cureus.48379

**Published:** 2023-11-06

**Authors:** Paige Stratton, Vithal Vernenkar, Aeryn J Fulton, Varun Soti

**Affiliations:** 1 Dermatology, Lake Erie College of Osteopathic Medicine, Elmira, USA; 2 General Surgery, Rochester Regional Health, Rochester, USA; 3 Immunology and Microbiology, Lake Erie College of Osteopathic Medicine, Elmira, USA; 4 Pharmacology and Therapeutics, Lake Erie College of Osteopathic Medicine, Elmira, USA

**Keywords:** buschke-lowenstein tumor (blt), cell-mediated immunity, immunity th2, aromatase inhibitor therapy, selective estrogen receptor modulator

## Abstract

Buschke-Lowenstein tumors (BLTs) are benign dermatologic manifestations of human papillomavirus (HPV). They originate from longstanding condylomata in individuals with compromised immune systems. In this case report, we present a 68-year-old immunocompetent female with HPV condylomata that had transitioned to a large, fungated BLT in her right groin. The patient's immunocompetency was determined by the absence of diabetes, corticosteroid therapy, organ transplant, cytotoxic therapy, or any known primary or other secondary immunodeficiencies. Notably, the patient had a history of breast cancer, managed through lumpectomy, local radiation, and two years of combined aromatase inhibitor and selective estrogen receptor modulator (SERM) therapy, followed by three years of further SERM therapy. We propose that the effect of her previously received SERM therapy shifted the T helper (Th)1 immune response to a Th2 response. This may have compromised the patient's HPV-specific cell-mediated immunity, favoring a non-protective Th2-dominant effect. Thus, it potentially enabled immune evasion, transitioning to a BLT phenotype. Additionally, the immune skewing of the SERM may have been initially opposed by the known ability of aromatase inhibitors to potentiate Th1 responses. Indeed, the patient first noticed the appearance of HPV condylomata progressing to the BLT phenotype with the cessation of the aromatase inhibitor therapy under the unopposed influence of the SERM. The resultant cytokine milieu may have contributed to the unusual progression to the BLT phenotype in this otherwise immunocompetent patient.

## Introduction

Human papillomavirus (HPV) is a well-researched virus with over 400 identified genotypes. It causes warts on the body's mucosal and cutaneous surfaces. Genotypes 6 and 11 of HPV are commonly associated with genital, peri-anal, and anorectal warts due to sexual contact [1]. Morphologically, these warts are usually small, less than 3 millimeters (mm) in size, and can range in color from flesh-colored to darkly pigmented. They tend to appear in clusters but may be solitary. While generally not painful, they may be pruritic. Their appearance can vary from verrucous to dome-shaped, smooth, rough, or flat plaques [2].

The Buschke-Lowenstein tumor (BLT), or giant condyloma acuminatum, is a benign dermatologic manifestation of infection with HPV genotypes 6 or 11. It is different from a typical condyloma primarily due to its size, although there is currently no defined classification system [3]. BLT presents as locally destructive invasive growth and does not resolve independently. Metastatic disease is rare but possible [4]. These tumors are usually slow-growing, taking an average of 2.8-9.6 years to develop, but they may grow faster in individuals with weakened immune systems [1].

BLT originates from an untreated, long-standing condyloma. It usually occurs in immunocompromised individuals. Cell-mediated immunity (CMI) plays a crucial role in controlling HPV-induced lesions, and when deficient, these growths can exceed 10 centimeters (cm) in size. There are multiple gene mutations involved in the development of BLT. The exact incidence of the disease is unknown, but reports estimate it to be approximately 0.1% in the general population, leading to limited accessible medical literature on the subject [5].

Although BLTs are benign, if they continue to grow unchecked, they can invade surrounding tissues and blood vessels, potentially causing destruction. On gross examination, BLTs typically present as sizeable, cauliflower-shaped growths. Histologically, they exhibit papillomatosis, hyperkeratosis, parakeratosis, acanthosis, and koilocytosis. To be classified as BLT, the histology must be negative for malignancy [1].

Nevertheless, BLTs have the potential to undergo malignant transformation. At diagnosis, approximately 56% of BLTs have transformed into squamous cell carcinoma (SCC) in situ. They are notoriously recurrent, with reported rates as high as 66% after excision. Other common complications associated with BLTs include secondary infections, which can lead to abscesses, fistula formation, and bacteremia, requiring chronic wound management [1].

Dermatologists treat BLTs through surgical and non-surgical methods, sometimes combining both modalities to reduce recurrence rates. The most widely accepted initial standard treatment is a wide radical excision of the lesion. The approach to wound healing varies among surgeons, with some preferring vacuum-assisted wound closure while others opt for flaps, grafts, or healing by primary or secondary intention alone [6].

## Case presentation

We present a case of a 68-year-old female (from whom signed consent was obtained for this case report) who presented to the emergency department with a large, fungated mass in the right groin accompanied by drainage and a putrid odor. The mass had been present for more than two years, but she noticed sudden inflammatory changes of two-month duration. Her medical history was significant for breast cancer, managed by a lumpectomy, local radiation, aromatase inhibitor (anastrozole, 1 milligram daily for two years), and a selective estrogen receptor modulator (SERM) therapy (tamoxifen, 20 milligrams daily for five years). She noted that the previously extant condyloma rapidly progressed during the treatment, with necrotic parts of the lesion detaching and falling off in the shower.

**Figure 1:**
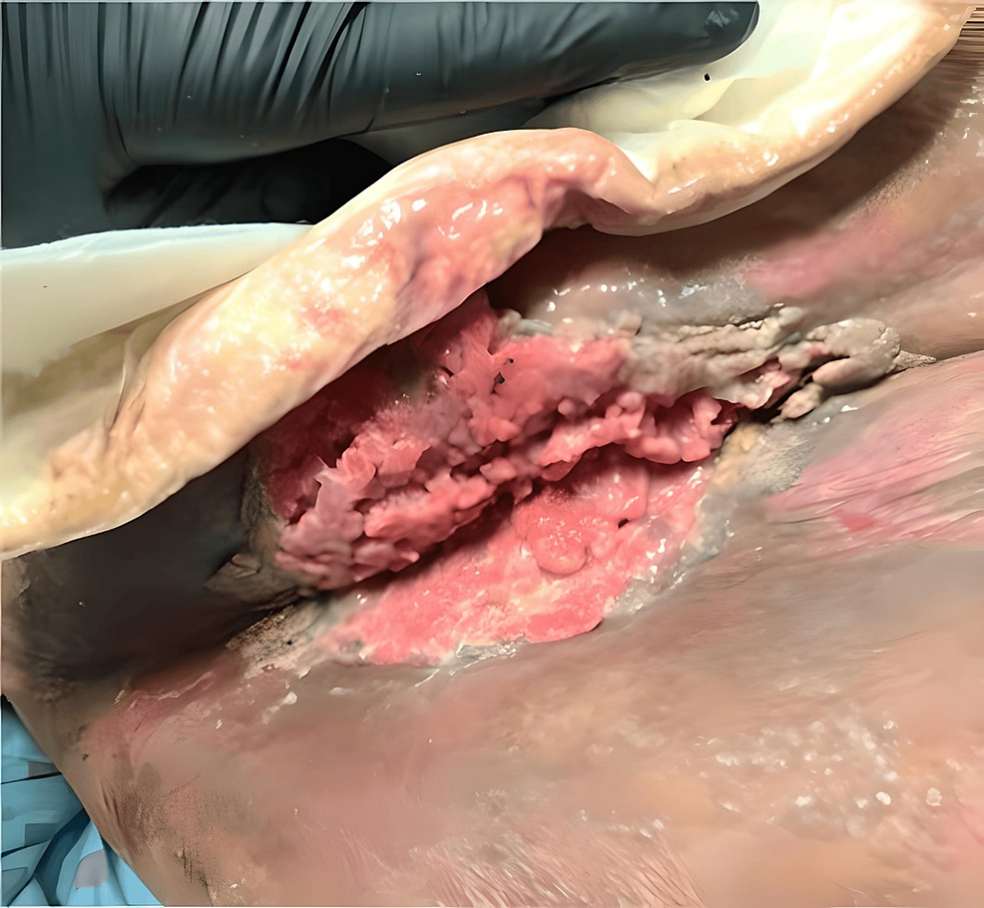
Exophytic growth with purulent fluid in the right groin. The figure shows a large fungating mass with purulent fluid discharge. The computed tomography scan correlated with the clinical findings, demonstrating the presence of an abscess beneath the fungating mass.

**Figure 2:**
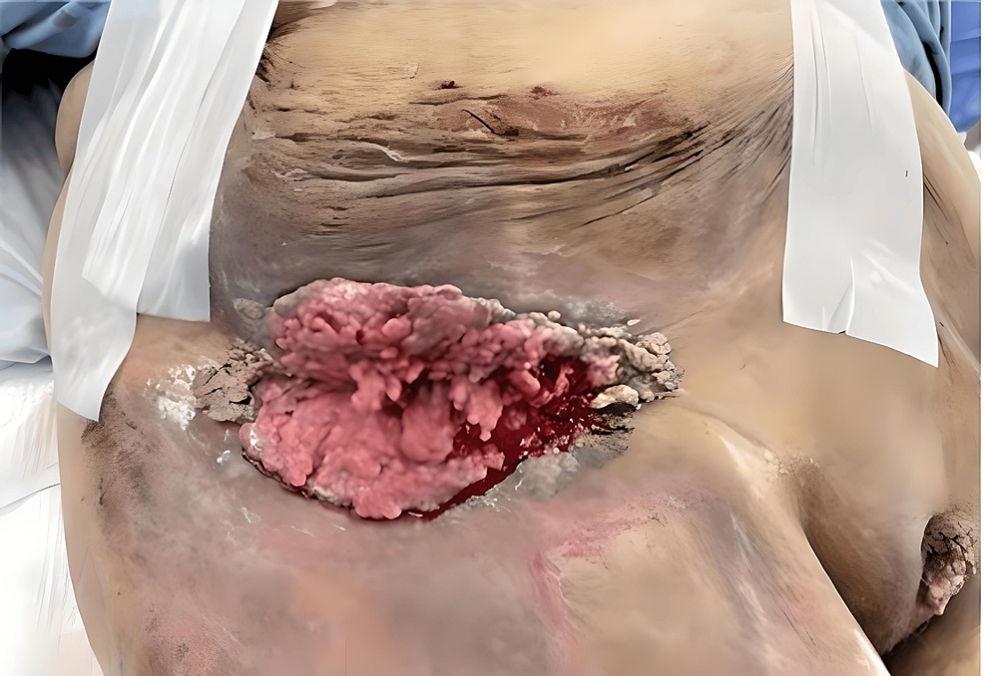
Fungating growth of the right groin with additional exophytic vulvar growth. The figure shows a pre-operative image of the right groin after thoroughly cleaning and prepping the area per surgical protocol. There was bleeding from the fragile tissue. Also shown is an additional growth in the vulvar area (in the lower right corner), which was not removed.

**Figure 3:**
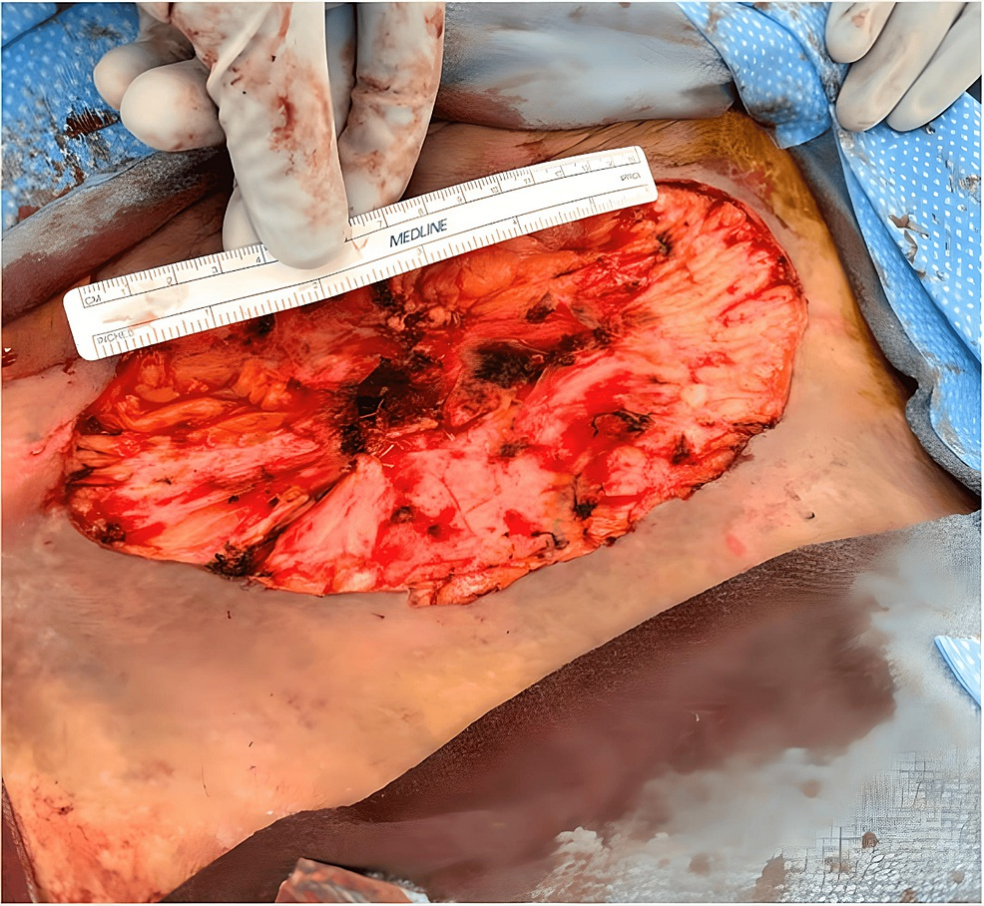
Post-surgical right groin area. The figure shows the right groin area after surgical wide excision of the exophytic mass. There was computed tomography evidence of an associated abscess. However, there was no gross abscess noted during the procedure. The area measured 20×15×3 centimeter post procedure.

**Figure 4:**
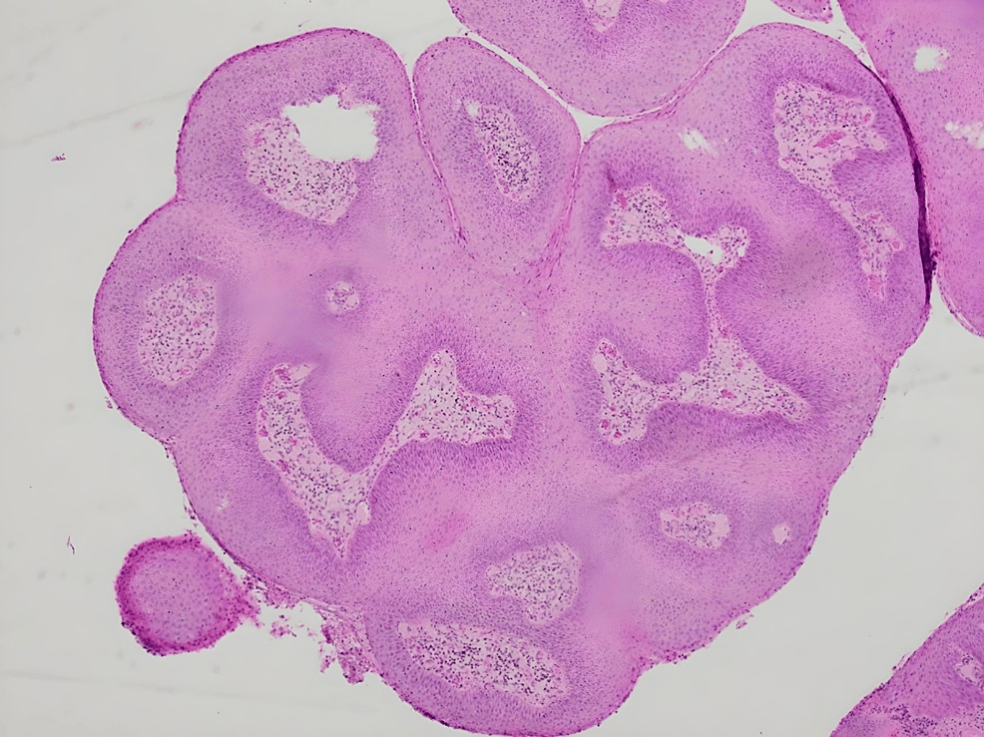
Histology slide demonstrating papillary squamous growth in a human papillomavirus-related lesion at 40× magnification. The histology slide (from a specimen taken from the right groin lesion) displays finger-like projections of the stratified squamous epithelium extending from the lesion's surface into the overlying epithelium. This histological feature is characteristic of human papillomavirus-associated lesions, aiding diagnosis and classification.

**Figure 5:**
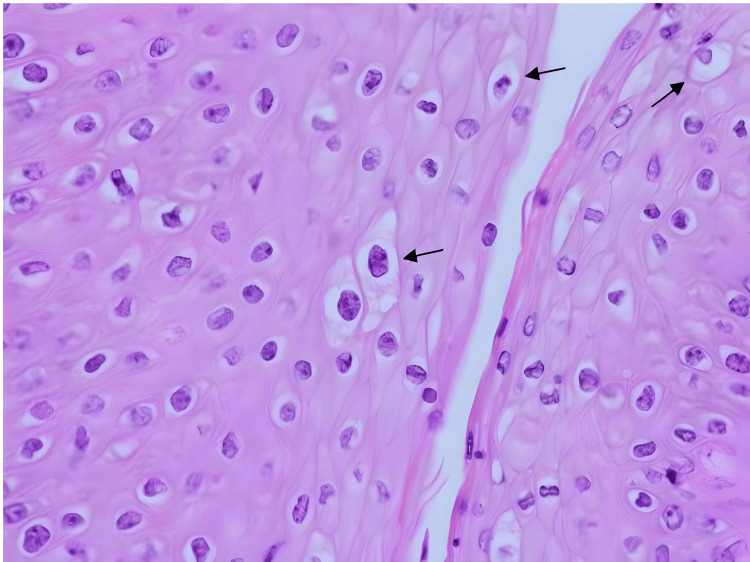
Histology slide demonstrating koilocytosis at 400× magnification. The histology slide (from a specimen taken from the right groin lesion) displays koilocytes, indicated by arrows. Koilocytes exhibit enlarged nuclei with hyperchromasia and perinuclear clearing, creating a characteristic halo-like appearance. It is a histological feature typical in human papillomavirus-associated lesions and aids in diagnosing human papillomavirus infection.

The patient underwent a computed tomography scan, which revealed an 8×3×5 cm abscess within the subcutaneous tissues of the right lateral pelvic wall (Figure 1 and Figure 2). A wide local excision removed the 20×15×3 cm mass (Figure 3). Post procedure, a negative pressure vacuum-assisted wound closure facilitated wound healing. Also notable was a distinct exophytic growth with cauliflower-like extensions protruding from the genital region, whose removal was deferred. Histological examination of the excised lesion demonstrated morphological findings consistent with HPV-associated lesions (Figure 4 and Figure 5).

The cultures of lesions removed tested positive for aerobic pathogens, including *Proteus mirabilis*, *Staphylococcus simulans*, and *Trueperella* species. Anaerobic cultures also yielded positive results for anaerobic gram-negative rods. The patient adhered to the appropriate antibiotic regimen and was discharged eight days after the procedure. After three weeks, a post-operative consultation determined to discontinue the negative pressure vacuum-assisted wound closure, and the patient was allowed to heal through secondary intention with weekly debridement.

## Discussion

BLTs were initially described by Felix Buschke and Paul Lowenstein in 1925. They reported a series of cases involving a rare wart-like lesion with a propensity for local invasion and recurrence. These lesions were distinguished by their morphology and had an increased risk of malignant transformation [7]. Detecting and managing BLTs early is crucial due to the high risk of malignancy. Effective management entails wide local excision, topical chemotherapy, or laser removal with phototherapy. Post-removal management is multidisciplinary, improving patient prognoses and quality of life [8]. A lack of consensus on grading and staging BLT results in delayed treatment and unfavorable outcomes.

There have been infrequent reports of putatively immunocompetent individuals, defined as those with no known diabetes, chronic disease, or corticosteroid treatment, afflicted with BLT [9]. The patient presented here was not immunocompromised. We suggest that SERM therapy and potentially aromatase inhibitor therapy may have facilitated her tumor progression by skewing a protective T helper (Th)1-dominant immune response to a Th2 phenotype [10]. While Th2-mediated humoral immunity is required to block the transmission of HPV to keratinocytes following inoculation, control of extant infection is believed to be mediated by Th1-driven CMI [11]. SERMs have been previously demonstrated to induce Th1 to Th2 immune skewing [10]. This is crucial as efficacious CMI is Th1-dependent, as showcased by the classic duality of protective Th1 vis-à-vis non-protective Th2 responses to numerous intracellular pathogens [12]. SERM-mediated immune skewing is worthy of further study, given the proven inhibitory effects of SERMs upon HPV-induced cervical neoplasia [13].

Anastrozole, an aromatase inhibitor, has been shown to enhance Th1 immune responses and decrease regulatory T-cell responses [14]. The patient's anastrozole therapy may have initially opposed the SERM-mediated immune skewing. Indeed, it was after the cessation of the aromatase inhibitor that the patient first noticed condylomata, which subsequently converted rapidly to the BLT phenotype concurrent with her remaining three years of SERM therapy.

## Conclusions

This case underscores the potential role of SERMs and aromatase inhibitor therapy on HPV condylomata. These factors may influence the immune response, shift CMI to humoral immunity, and contribute to the development and progression of BLTs in otherwise immunocompetent patients. Understanding the interplay between sex steroid modulators and HPV lesions is vital for effective management and treatment strategies. Further research is needed to explore the complex relationship of these medications with efficacious immunological responses to HPV condylomata and their potentiation of BLT phenotypic progression.
